# Supplementary material for: Single-Shot Local Injection of Microfragmented Fat Tissue Loaded with Paclitaxel Induces Potent Growth Inhibition of Hepatocellular Carcinoma in Nude Mice
Source: Cancers (Basel). 2021 Nov 2;13(21):5505. doi: 10.3390/cancers13215505 (PMC8583409; doi:10.3390/cancers13215505)
Supplement: Supplementary file 1 [file cancers-13-05505-s001.zip › cancers-1435179-supplementary.pdf]

Supplementary Material and Methods

Sample collection, ethics statements, MFAT and DMFAT preparation

Samples of LP were obtained by liposuction of subcutaneous tissue as previously described elsewhere by using disposable cannulas provided with the Lipogems® kit [9, 10]. Tissue samples were collected from plastic surgery operations after signed informed consent by the patient, in accordance with the Declaration of Helsinki. The approval for their use was obtained from the Institutional Ethical Committee of Milan University (n.59/15, C.E. UNIMI, 09.1115). For all the *in vitro* and *in vivo* experiments performed in this study fat tissue was obtained from five different human donors that underwent plastic surgery.

MFAT specimens were obtained as previously described [9, 11]. Briefly, by using a standard 225-ml Lipogems® device (provided by Lipogems® International, Milan, Italy), LP collected by syringe was pushed into the Lipogems® device through a filter for a first clusters reduction and complete disaggregation was obtained by shaking the device containing inside five stainless steel marbles. Afterwards, the micro fragmented fat tissue was aspirated by a syringe connected with the device and was ready for experiments. DMFAT was prepared following a previously published procedure [8]. Briefly, aliquots of MFAT (5 ml) were transferred in a conical tube washed with PBS three times by centrifugation at 200 g × 10 minutes. After discarding PBS washing solution, MFAT undergoing a freeze (−20°C) and thaw (F/T) cycles (usually three of 30 minutes each) that lead to the killing of all the cells in the stromal vascular fraction (SVF). Aliquots of DMFAT (1–2 ml) were kept at −80°C until use for experiments, others were analyzed to verify the absence of cell vitality by SVF extraction with collagenase (SIGMA St. Louis, MO, USA) (0.2% w/v) following processing the final cell pellets by Tripan-blue assay. To note that DMFAT specimens before used for in vitro and in vivo experiments were washed several times with PBS by centrifugation in order to remove as much as possible all cell debris and the residual presence of membrane proteins and genetic material content and then investigated as previously described [12].

Evaluation of the activity of MFAT and DMFAT loaded with PTX on Hep-3B growth in 2D assay

The anti-cancer activity of CM derived from both MFAT-PTX or DMFAT-PTX specimens was evaluated in a 72-hour proliferation assay as previously described [8, 13]. Briefly, around 2×10^4 Hep-3B cells were seeded in wells (24-multiwell plate) and then covered with 500 µl/well of complete MEM medium and left to adhere 3 hours. Then, CM at different dilutions (from 1:2 to 1:10), derived from cultured MFAT-PTX, DMFAT-PTX and from control untreated specimens, were added to wells and further incubated for 72 hours. At the end of incubation cancer cells were detached with trypsin and counted as previously described [13]. The anti-tumor activity of CM from MFAT-PTX and DMFAT-PTX were compared to the one of pure PTX and expressed as PTX equivalent concentration (p-EC) according to the following algorithm p-EC (ng/ml) = IC_{50} PTX x 100/V_{50} (µl/well) where IC_{50} PTX is the concentration of pure PTX producing 50% growth inhibition and V_{50} the respective volume of CM that produces the same inhibition.

Histological analyses of DMFAT – Hep-3B cells in 3D constructs

The efficacy of MFAT-PTX or DMFAT-PTX specimens on Hep-3B was also investigated in a 3D assay. Briefly, 50 µl of control or PTX loaded MFAT specimens were mixed at 4°C with 100 µl of Matrigel (BD Biosciences, Franklin Lakes, NJ, USA) where Hep-3B (3 and 5 x 10^6) cells were added and left to jellify for one hour at 37°C. Then, complete growth MEM was added to gels and further incubated for 72 hours. At the end, medium was removed, and gel processed by immunocytochemical analysis through cyto-inclusion technique [14]. Samples were fixed in 4% paraformaldehyde (PFA) and cryoprotected overnight at 4°C by immersion in a 30% (wt/vol) sucrose solution before being embedded in Tissue-Tek O.C.T. Compound (Tissue-Tek; Sakura Finetek, Torrance, CA, www.sakuraus.com.) and frozen. Sections were cut 5-µm thick with a cryostat at -20 °C and stained with H&E (Sigma-Aldrich, St. Louis, MO, USA) or Hoechst 33342 (Thermo Fisher Scientific) to detect apoptotic cells. Samples were visualized using conventional light or fluorescent microscopes. In other series of experiments apoptosis was investigated by using Annexin V staining

(Thermo Fisher Scientific). Briefly, after 72 hours of incubation, Hep-3B cells were extracted from 3D constructs by digestion with collagenase (Sigma). After cells washing by centrifugation, Fluorescent Annexin V conjugates was used in flow cytometry as previously described (6).

Evaluation of in vivo anti-tumor activity of DMFAT-PTX

Five-week-old athymic nude-Foxn1nu mice were purchased from Envigo (Envigo, Bresso, Italy) and were housed under pathogen-free conditions. Experiments were reviewed and approved by the licensing and ethical committee of IZSLER (Istituto Zooprofilattico Sperimentale della Lombardia e dell' Emilia Romagna, Brescia, Italy) and by the Italian Ministry of Health, and in compliance with the "ARRIVE" guidelines (Animals Research: Reporting in Vivo Experiments).

In a preliminary experiment, 3 mice were injected sc with 5×10^6 Hep-3B cells in 100 ul of MTG in the right flank. Mice were observed daily to establish the day of tumor appearance and when the nodule was 1 cm in diameter, mice were sacrificed, and tumor was removed and investigated to verify the HCC features by histological examination. To note, for these experiments only DMFAT specimens were used, because of the advantage given by the possibility to conserve frozen biomaterials until use in mice.

In the first series of experiments, mice (n=6/group) were injected with 5×10^6 Hep-3B cells in the right flank (day 0). The tumors were allowed to grow to an average 0.5/0.7 cm in diameter corresponding to a tumor volume ranging from 65 to 179 mm^3 (median weight 120 mg) that were calculated using the formula $1/6\pi d^3$ (13). After 10-14 days the mice were randomly subdivided into 4 groups and treated just next to the tumor nodule with a single shot of 200 µl saline (control group CTRL), DMFAT (200 µl), DMFAT-PTX 10 mg/kg (200 µg/200 µl) and free PTX drug (200 µg/200 µl saline), respectively. Loading of PTX into just thawed DMFAT specimens were performed 20-30 minutes before injection as described above, adding 1 mg of PTX (around 166 µl of the stock PTX solution 6 mg/ml) to 1 ml of DMFAT and agitation. After treatments, mice were observed daily; every two days tumor diameters were measured by caliber. According to the ethical protocol, all the mice were sacrificed when the tumor nodule reached 2.0-2.5 cm in diameter (≥ 2 g of weight) or, whatever the diameter, sacrificed on day 60 after the transplant. At this time only tumor-free mice were followed until 90 days. Animals were euthanized with carbon dioxide inhalation, followed by cervical dislocation. In a second series of experiments mice (n=6/ group) were similarly injected s.c. with 5×10^6 Hep-3B and treated locally with half dose of PTX, DMFAT-PTX (100 µg PTX/200 µl) corresponding to 5 mg/kg.

PK of PTX released by DMFAT-PTX in vivo

For the PK and biodistribution experiment, five-week-old athymic nude-Foxn1nu mice were purchased from Envigo (Envigo, Bresso, Italy) and were housed under pathogen-free conditions. Experiments were reviewed and approved by the licensing and ethical committee of IZSLER (Istituto Zooprofilattico Sperimentale della Lombardia e dell' Emilia Romagna, Brescia, Italy) and by the Italian Ministry of Health. Normal and tumor bearing mice were injected sc (n = 3) with 5 mg/kg of DMFAT-PTX (corresponding at 100 µg PTX in 200 µl of DMFAT). Two, 24, 48 72 and 168 (7 days) hours post treatment, blood together with the site of DMFAT-PTX injection from normal and tumor bearing mice treated sc were collected and stored until use.

Plasma extraction procedure

Extraction and purification from plasma was performed by SPE. 50 µL plasma was added with 100 µl of IS (PTX D5 0.1 µg/mL) and 850 µL of water, then sonicated for 30 min at 40°C (Sonorex, Bandelin electronic, Berlin). Samples were centrifuged for 5 minutes at 10000 rpm (MiniSpin, Eppendorf, Hamburg). Solid-phase extraction was performed on Strata TM-X 33 µm Polymeric Reversed Phase SPE 30 mg/1 mL extraction cartridges from Phenomenex (Anzola Emilia, Italy) connected to Visiprep Solid Phase Extraction Vacuum Manifolds from Supelco (Bellefonte, USA). Before use, the cartridges were conditioned with 1 ml methanol and 1 ml deionized water. The diluted samples were percolated through the cartridges. The cartridges were then rinsed with 1 mL deionized water with 5% meOH, and vacuum-dried for 5 minutes to remove excess water. Finally, the retained compounds were eluted with 1 ml of methanol/isopropanol/formic acid (60:39.2:0.8) and the elution was collected in a

test tube. The eluent was evaporated until dryness by a gentle nitrogen stream. Finally, the residue was re-dissolved with 150 µl acetonitrile and 10 µl were injected for HPLC coupled to a tandem mass spectrometer (LC–MS/MS) analysis.

Tissue extraction procedure

Extraction and purification from different tissues (sc injection area, and tumor sample) was performed by single-step extraction. Weighted tissues (10-50 mg) were homogenized in 100 µl of methanol by TissueLyser LT (Qiagen, Hilden, Germany) for 3 at 50 oscillations/s. Samples were added with 100 µl of IS (Paclitaxel D5 0.1 µg/mL) and 800 µl of methanol/isopropanol/formic acid (60:39.2:0.8), then sonicated for 30 min at 40°C. The extract was evaporated until dryness by a gentle nitrogen stream. The tissues residue was re-dissolved with 150 µl of methanol, centrifuged for 10 minutes at 10000 rpm, filtered through a NY 0.45 µm filter (LLG labware, Meckenheim) and transferred in a vial and 10 µl were injected for LC–MS/MS analysis.

LC-MS/MS conditions

The analytical system consisted of a HPLC coupled to a tandem mass spectrometer. The liquid chromatograph system was a Dionex 3000 UltiMate instrument with autosampler, binary pump and column oven (Thermo Fisher Scientific, MA, USA). Separation was attained on a reversed-phase Luna C18(2) 50 mm x2, 3 µm particle size (Phenomenex, CA, USA) analytical column, preceded by a security guard cartridge with a linear gradient between eluent A (water + 5 mM ammonium formate + 0.1% formic acid) and eluent B (acetonitrile + 0.1% formic acid). The column was equilibrated with 20% (B) for 2 minutes, increased to 95% (B) in 4 minutes, held for 0.5 minutes, back to the initial conditions in 0.5 minutes and kept for 2 minutes at 20% (B). The flow rate was 0.4 ml/minute, the autosampler and the column oven were kept at 15°C and 30°C, respectively. The tandem mass spectrometer was an AB Sciex 3200 QTRAP instrument with electrospray ionization TurboIonSpray™ source (AB Sciex S.r.l., Milano, Italy). Instruments were managed with the proprietary manufacturer's software and according to the manufacturer's instructions. The analytical data were processed using Analyst software (version 1.6.2). The ion spray voltage was set at 5.5 kV and the source temperature was set at 400°C. Nitrogen was used as a nebulizing gas (GS 1, 40 psi), turbo spray gas (GS 2, 45 psi) and curtain gas (30 psi). The collision-activated dissociation (CAD) was set to a medium level. The dwell time was set at 0.3 s, and the MS scan was performed in positive ion modes (ESI+). The product ion spectrum (MS–MS) was generated at optimized DPs to identify the prominent product ions of the analytes using nitrogen as the collision gas. The collision energies (CE) of product ions transition were optimized by CE ramping via direct infusion. Multiple reaction monitoring (MRM) mode was used. In table 1 below are reported the optimal compound-dependent parameters (**Suppl. Table 1**).

Performance of the analysis of PTX by LC-MS/MS

The analytical system for PTX quantification consisted of a HPLC coupled to a tandem mass spectrometer (**Suppl. Fig. 9–11**).

Method validation: linearity and LOQ

The linearity was proven according to the regression line by the method of least squares and expressed by the coefficient of correlation (R2). Six-point matrix-matched calibration curves were evaluated by spiking increasing amounts of the analyte in blank plasma. Calibration curves were obtained by plotting the ratio between the peak area of the quantifier ion of the analyte and the peak area of the quantifier ion of the internal standard versus the corresponding concentrations of the analyte in concentration range between 0 and 100 ng/vial. Linearity was observed in the whole range. The values of the correlation factors R2 of the calibration curves were higher than 0.99. The LOQ values obtained was 0.5 ng/vial calculated by Multiquant software 2.1 at an accuracy between 80-120% and CV%<20%. Recovery from different biological matrices with the two extraction methods ranged from 63 to 70 % (Suppl. Fig. 11).

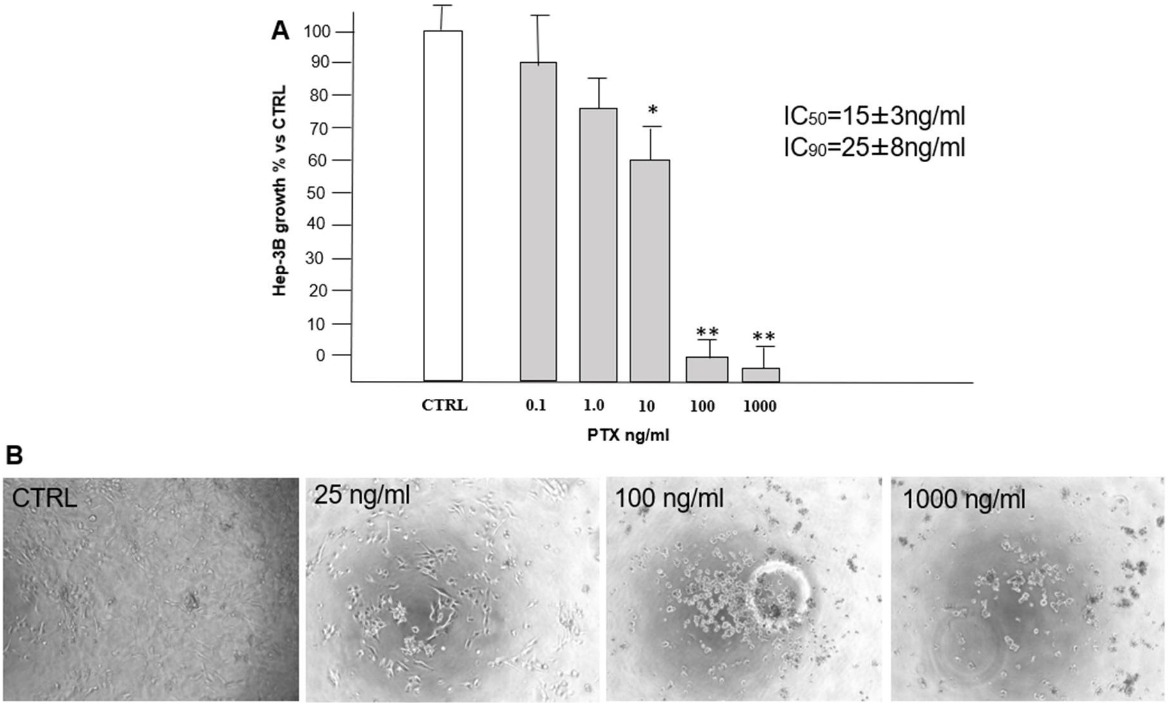

Figure S1. PTX displayed anti-proliferative activity on Hep-3B cells in culture.

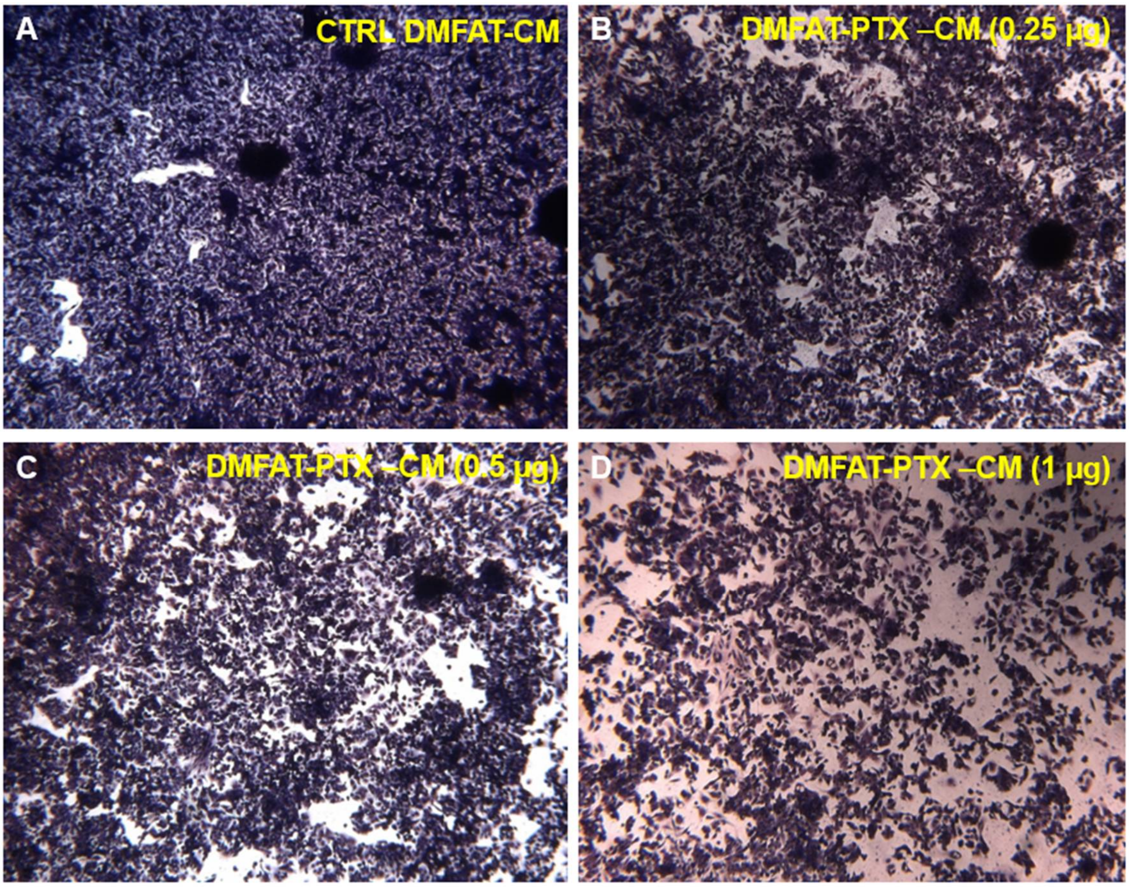

Figure S2. Effect of DMFAT-PTX-CM on Hep-3B growth.

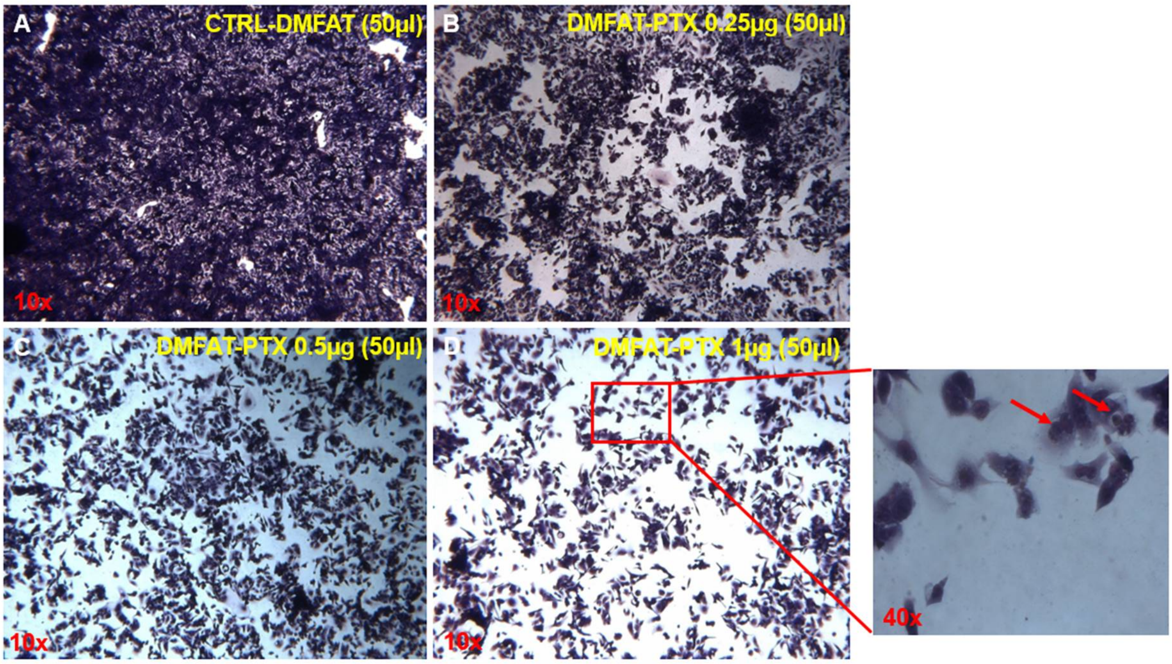

Figure S3. Effect of DMFAT-PTX specimens (50 μL) placed in the upper well of a trans-well insert.

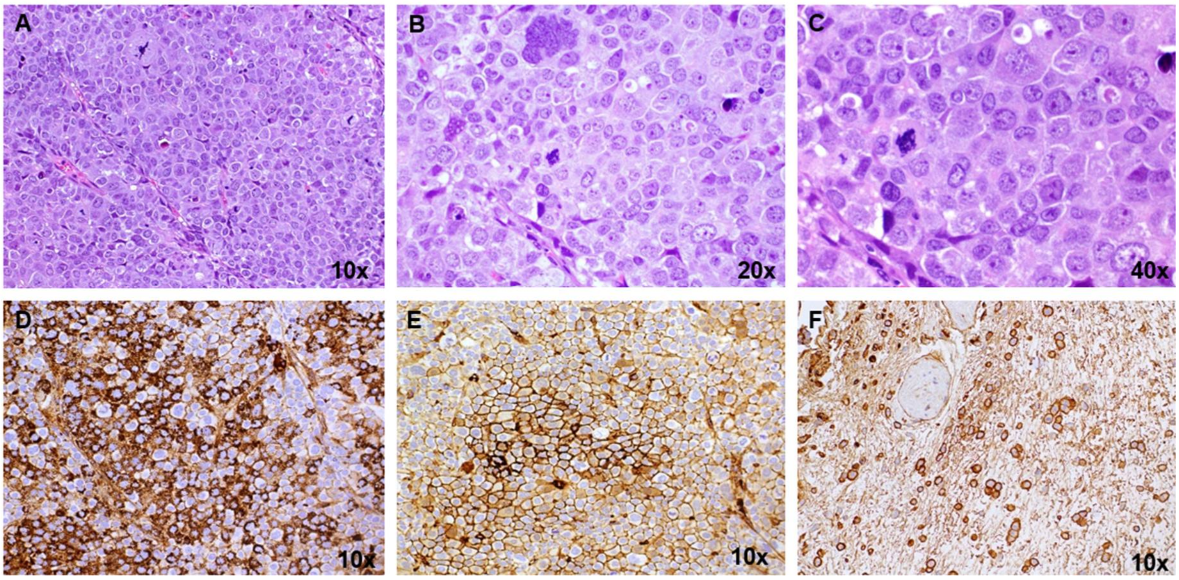

Figure S4. Immunohistochemical characterization of Hep-3B-induced HCC in mice.

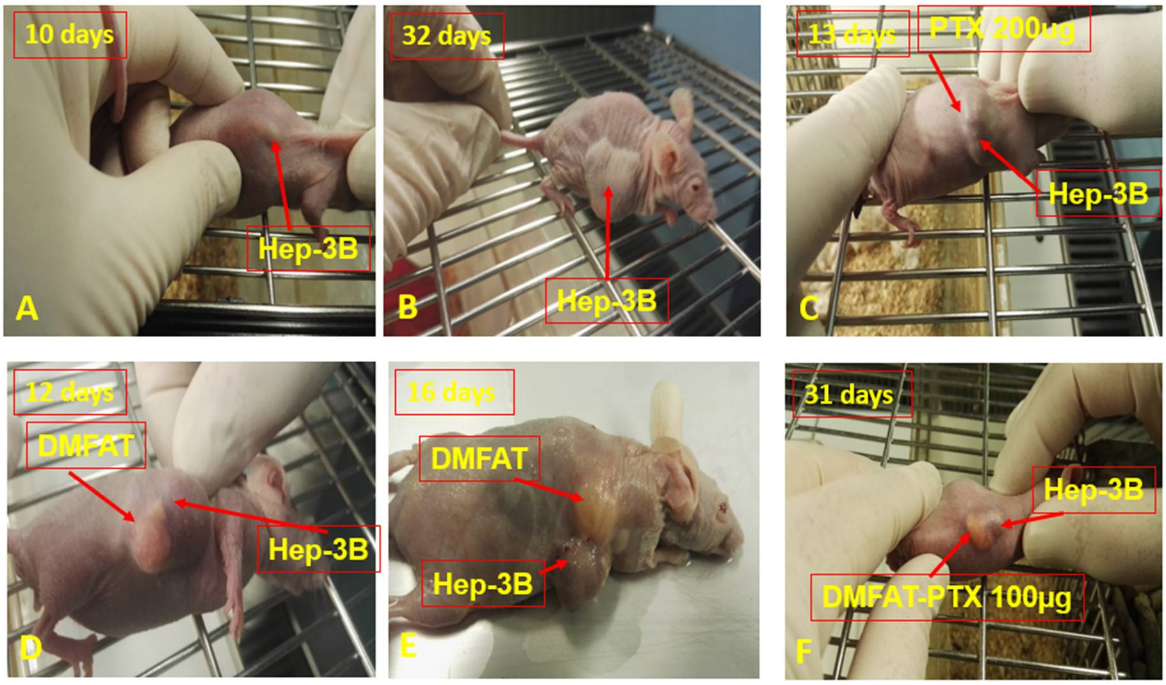

Figure S5. Representative photographs of mice injected in the right flank with Hep-3B cells and treated with DMFAT loaded or not with PTX.

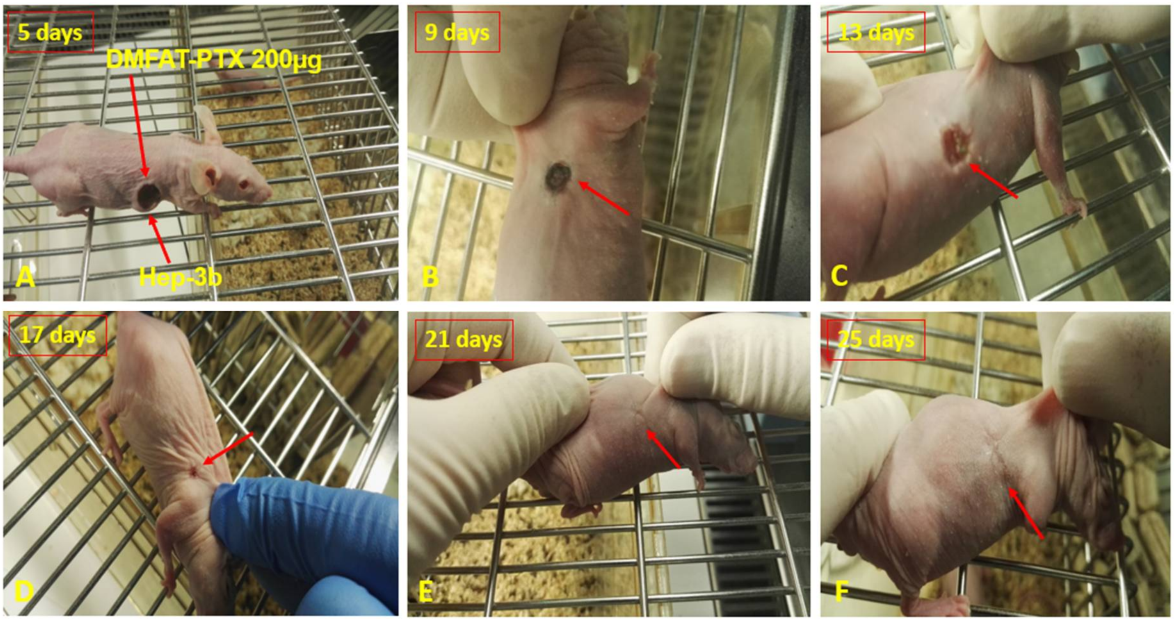

Figure S6. Local injection of DMFAT-PTX (200 µg/200 uL) induced Hep-3B regression but also skin lesions that healed spontaneously.

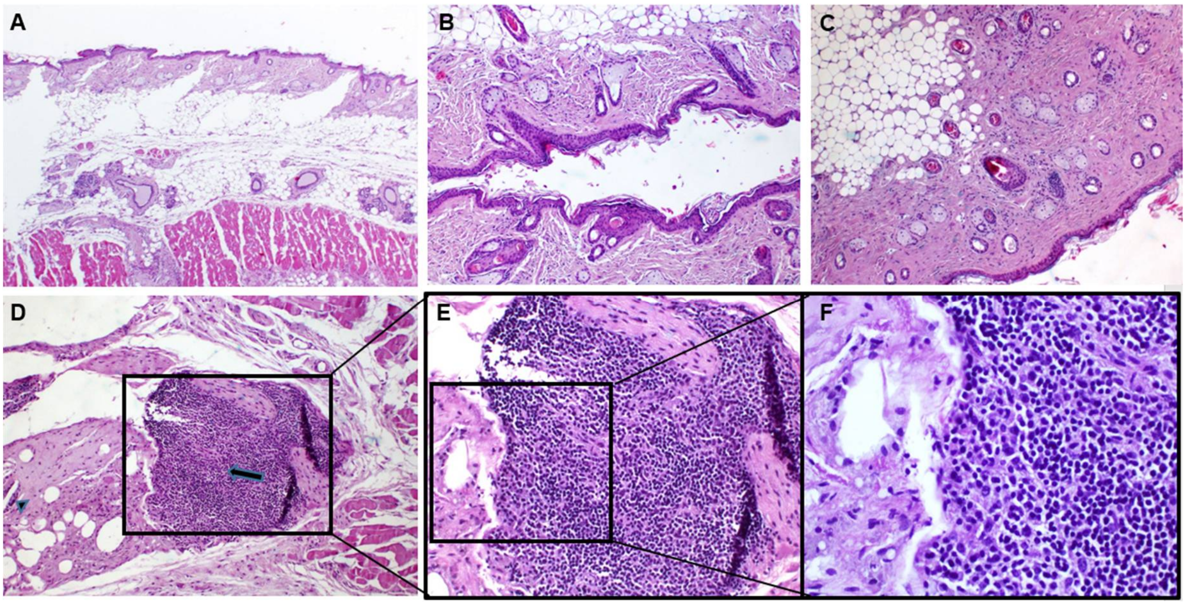

Figure S7. Skin histology of mice (90 days) affected by the HCC tumor when cured.

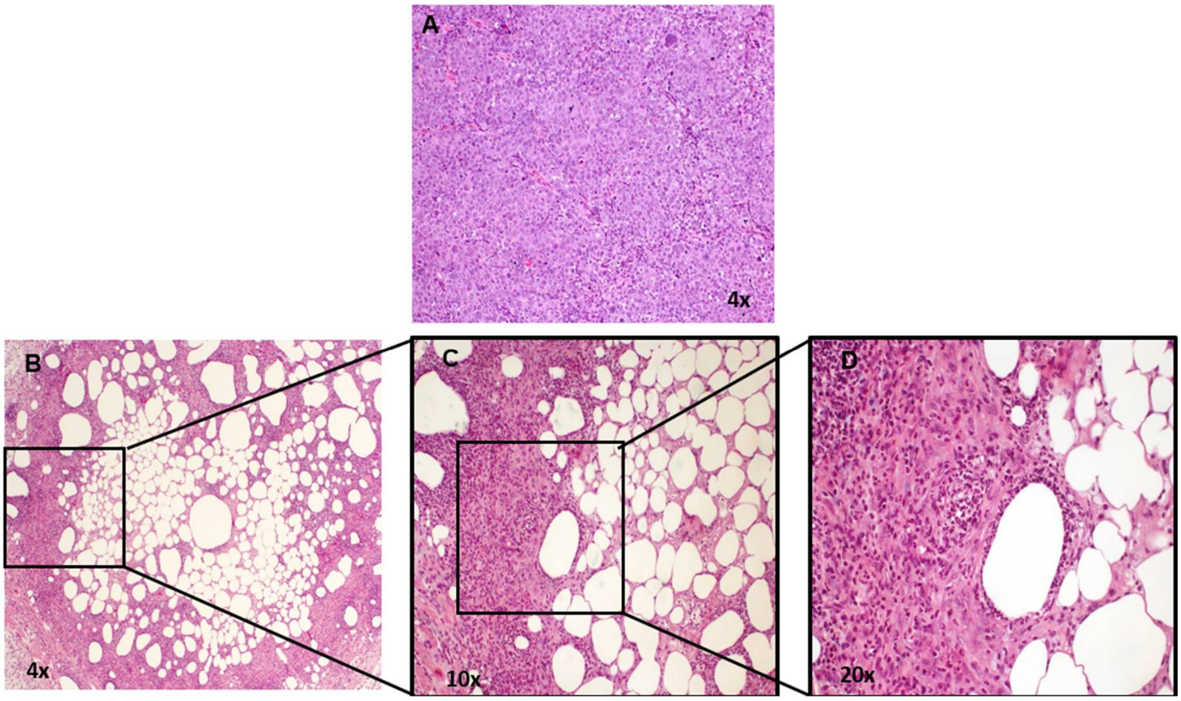

Figure S8. Histopathological analysis of the HCC tumor induced by Hep-3b cells treated with DMFAT.

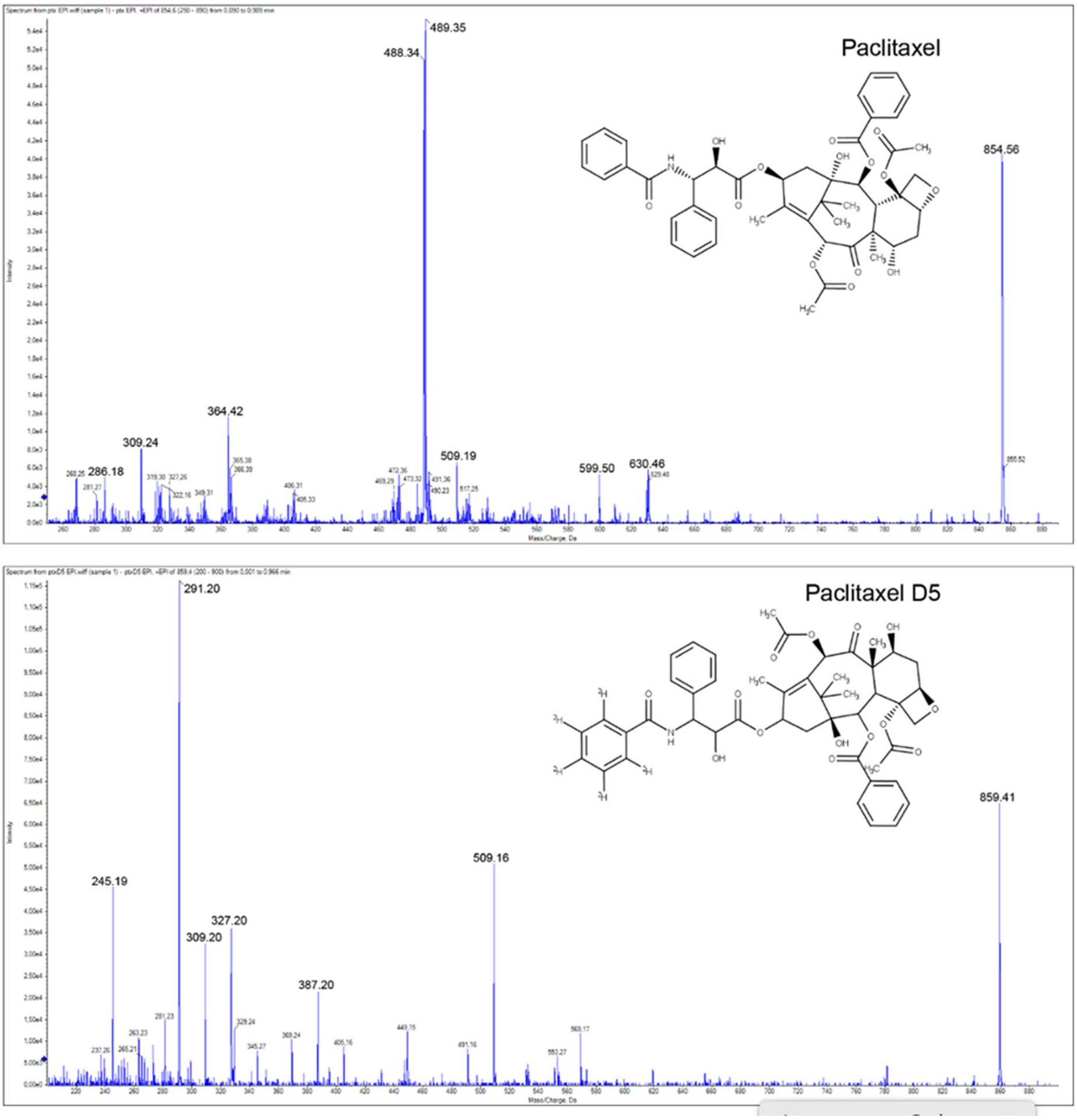

Figure S9. MS-MS spectra of paclitaxel and paclitaxel D5 obtained via direct infusion.

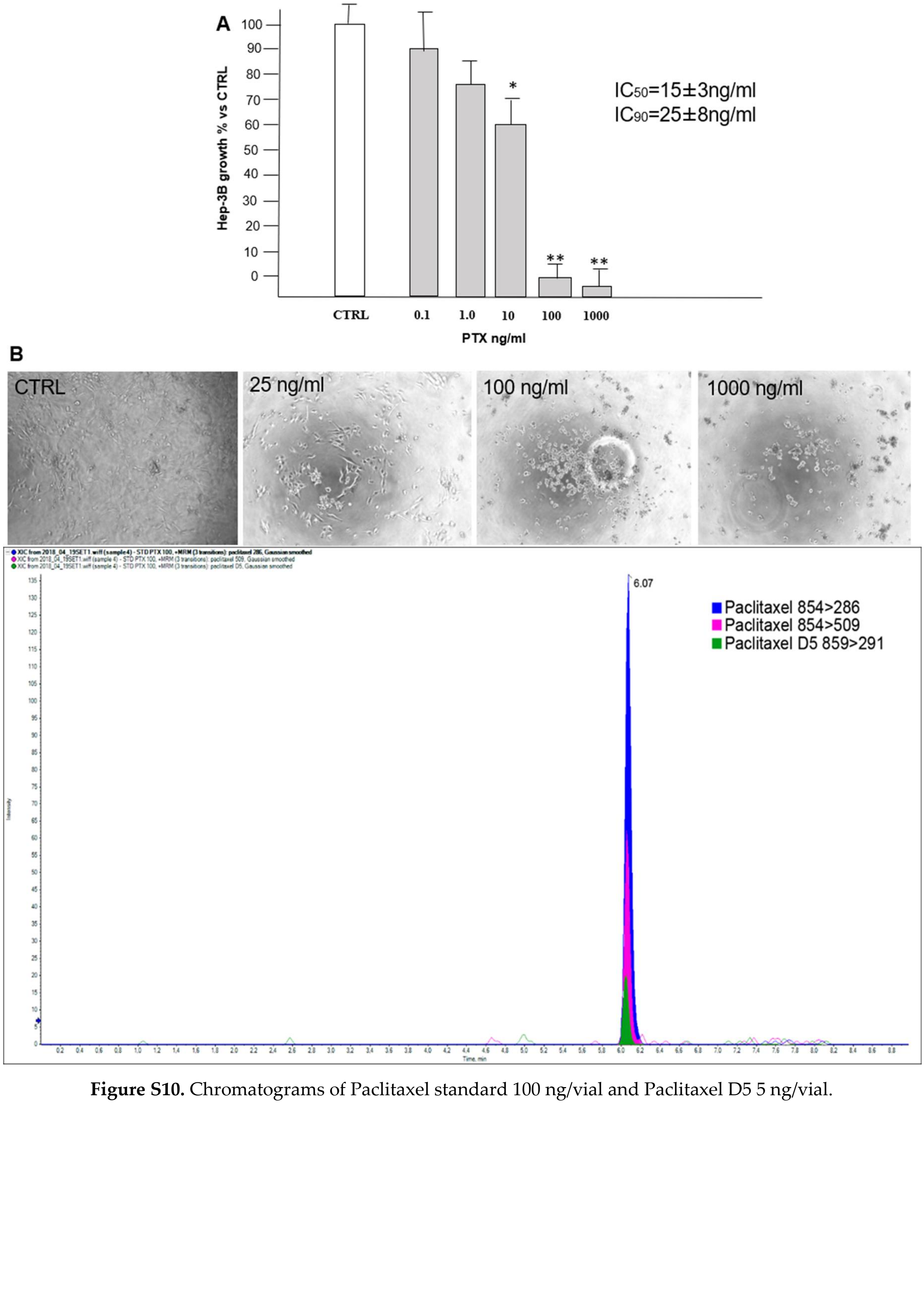

Figure S10. Chromatograms of Paclitaxel standard 100 ng/vial and Paclitaxel D5 5 ng/vial.

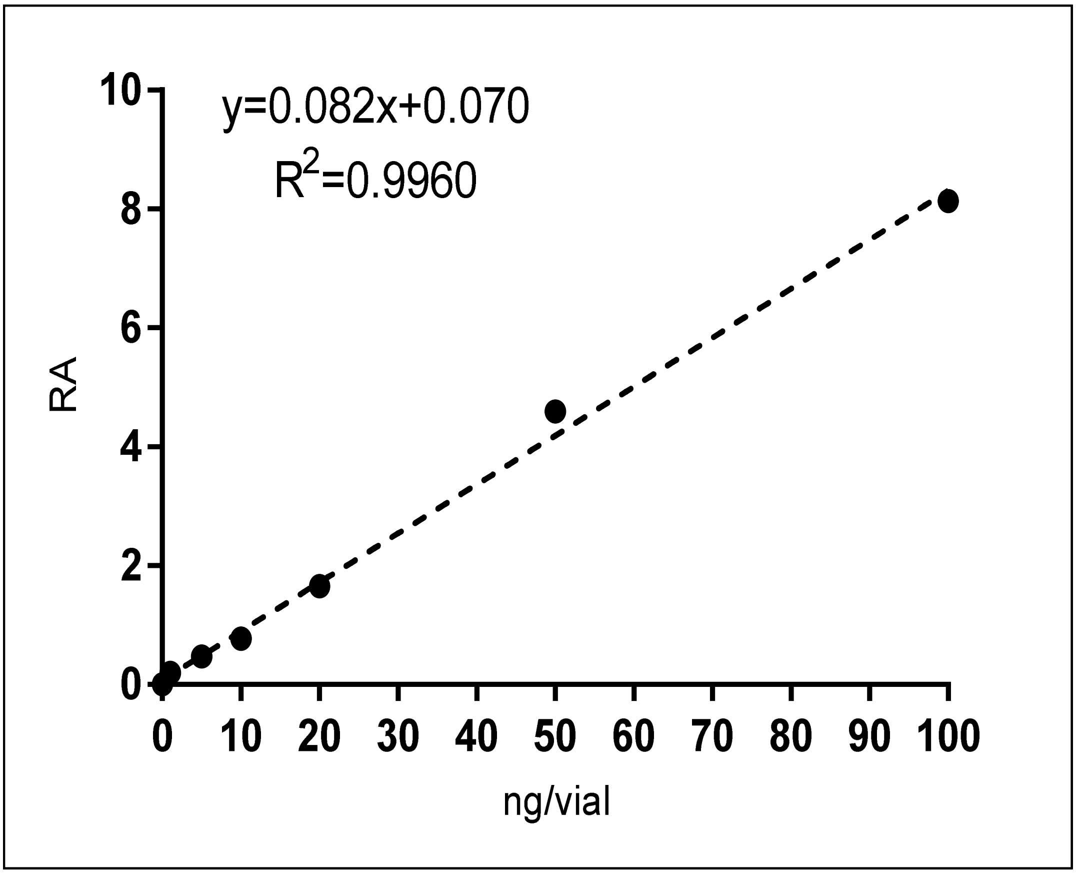

Figure S11. Paclitaxel plasma calibration curve.

Table S1. MS condition for each analyte, in bold transition used for quantification.

Analytes	Transition	DP (eV)	EP (V)	CE (V)
Paclitaxel	854.5 > 286.1	28	10	27
	854.5 > 509.0	28	10	17
Paclitaxel D5 (IS)	859.4 > 291.5	28	10	19
